# Supplementary material for: Inflating hollow nanocrystals through a repeated Kirkendall cavitation process
Source: Nat Commun. 2017 Nov 2;8:1261. doi: 10.1038/s41467-017-01258-0 (PMC5665896; doi:10.1038/s41467-017-01258-0)
Supplement: Supplementary file 1 — Supplementary Information [file 41467_2017_1258_MOESM1_ESM.pdf]

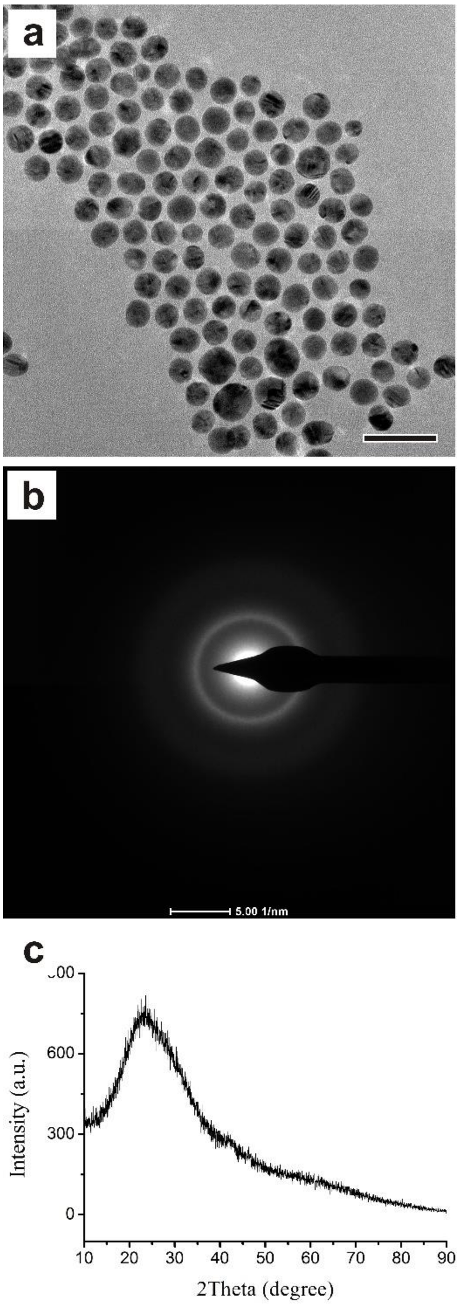

Supplementary Figure 1. Characterization of the amorphous Pd-P intermediates. (a) TEM image, (b) selected area electron diffraction (SAED) pattern and (c) XRD pattern. Scale bar in (a) is 50 nm.

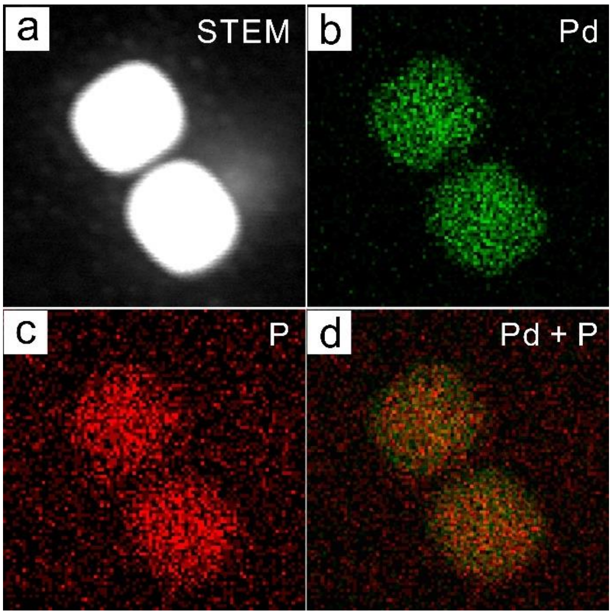

Supplementary Figure 2. EDX elemental mapping results for PdP_2 intermediates, suggesting that both Pd and P are homogeneously distributed in the PdP_2 intermediates. (a) STEM image, (b) distribution of Pd (green), (c) distribution of P (red), and (d) overlap of Pd and P.

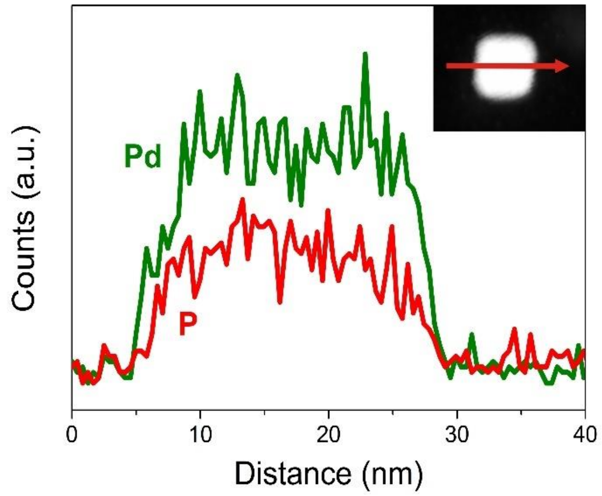

Supplementary Figure 3. HAADF-STEM image and cross-sectional compositional line profiles of a PdP_2 nanoparticle.

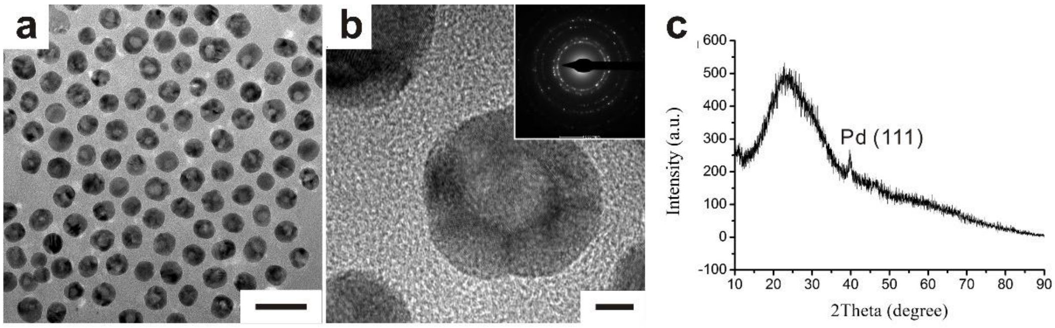

Supplementary Figure 4. Characterization of the prepared hollow Pd nanocrystals. (a) TEM image, (b) HRTEM image, and (c) XRD pattern. The inset in (b) shows the SAED pattern of the hollow Pd nanocrystals. Scale bar in (a) is 50 nm. Scale bar in (b) is 5 nm.

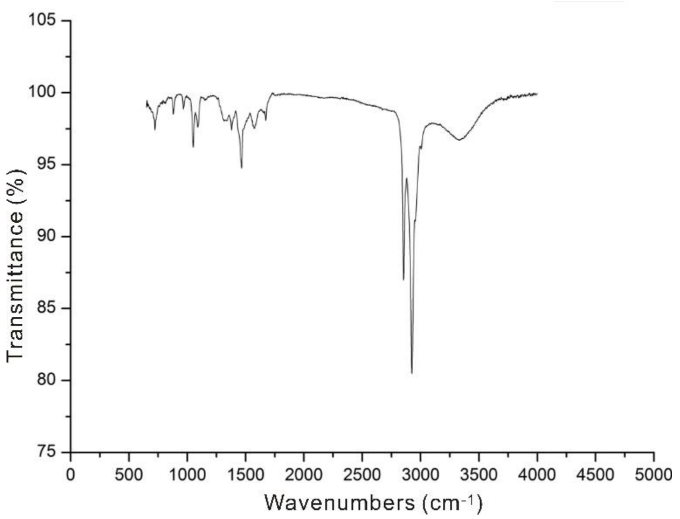

Supplementary Figure 5. FT-IR spectrum of H-Pd-3 hollow nanoparticles after washing with ethanol.

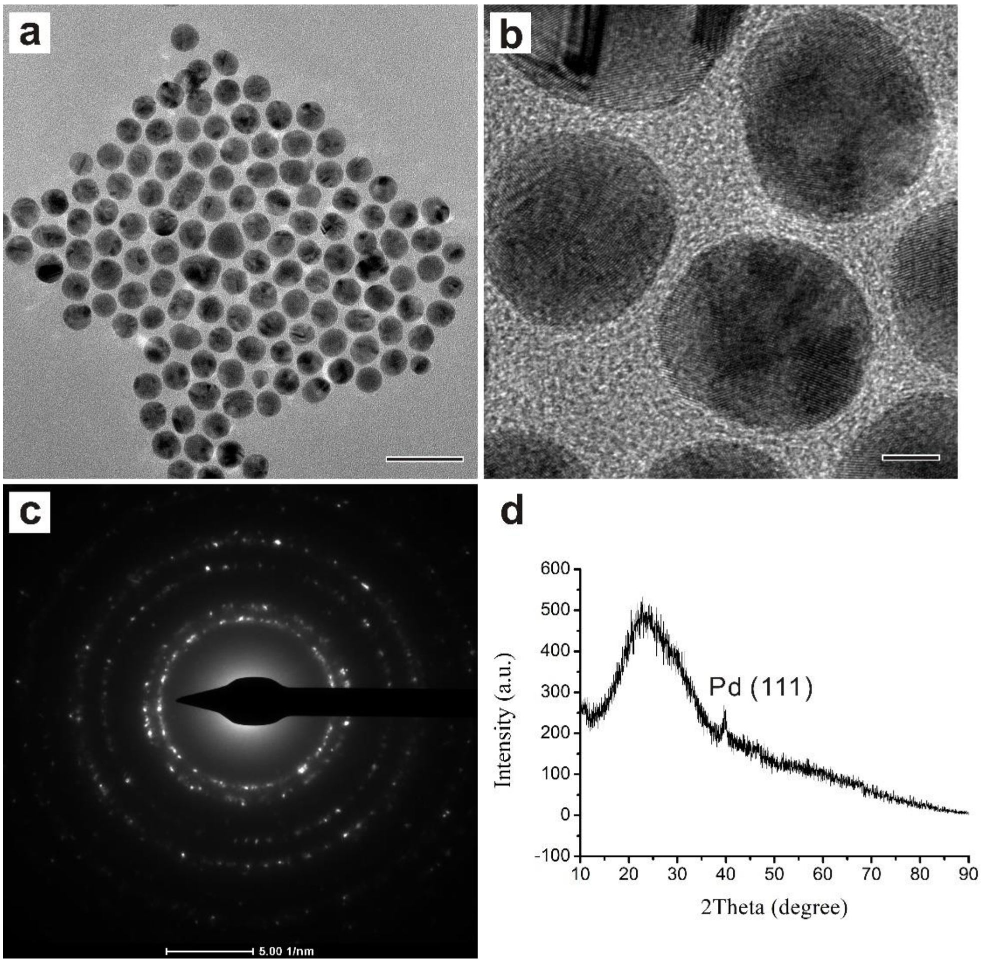

Supplementary Figure 6. Characterization of solid Pd spheres prepared under the protection of N_2. (a) TEM image, (b) HRTEM image, (c) SAED pattern, and (d) XRD pattern. Scale bar in (a) is 50 nm. Scale bar in (b) is 5 nm.

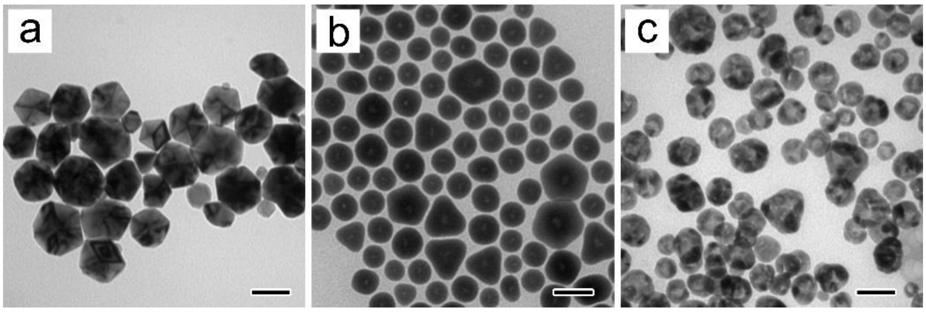

Supplementary Figure 7. Transformation of Pd decahedrons into hollow Pd nanocrystals. (a-c) TEM images of Pd decahedrons, Pd-P intermediates, and hollow Pd nanospheres, respectively. Scale bars in (a-c) are 50 nm.

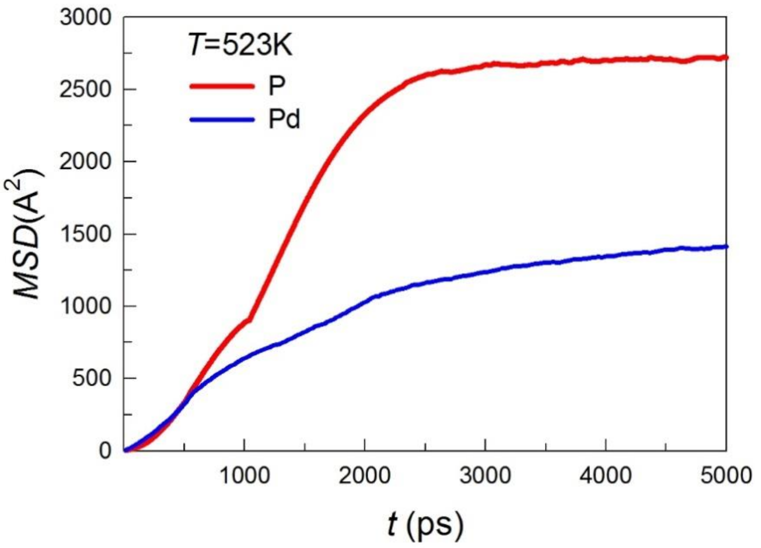

Supplementary Figure 8. Time-dependent MSD of different element types at 523 K.

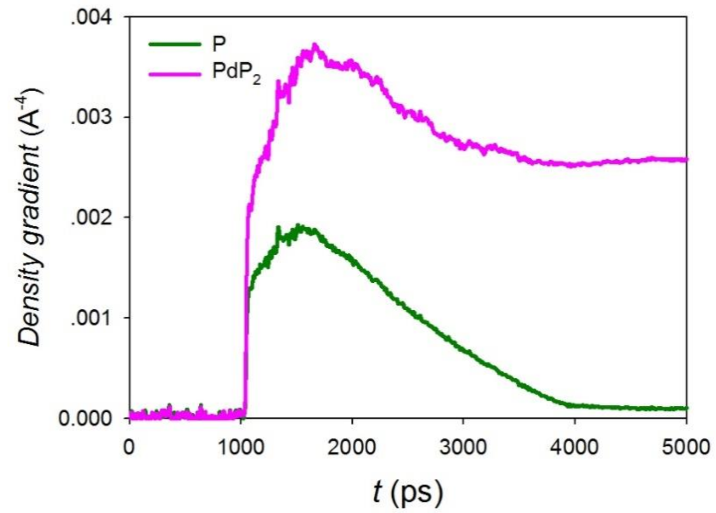

Supplementary Figure 9. Atomistic concentration gradient as a function of simulation time t at 523 K.

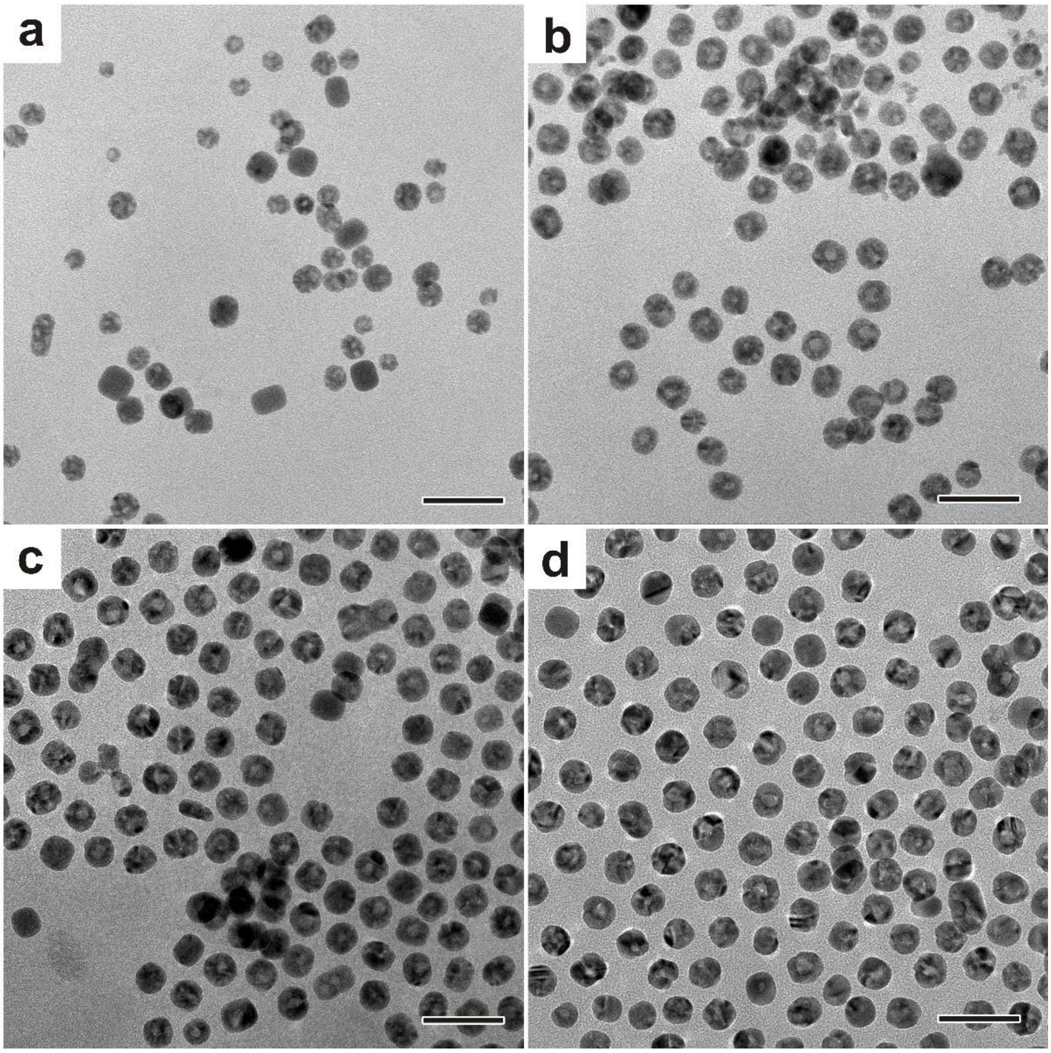

Supplementary Figure 10. Influence of the temperature during the formation of hollow Pd nanocrystals. TEM images of the products obtained at different temperatures: (a) 150 °C, (b) 180 °C, (c) 200 °C, and (d) 230 °C. Scale bars in (a-d) are 50 nm.

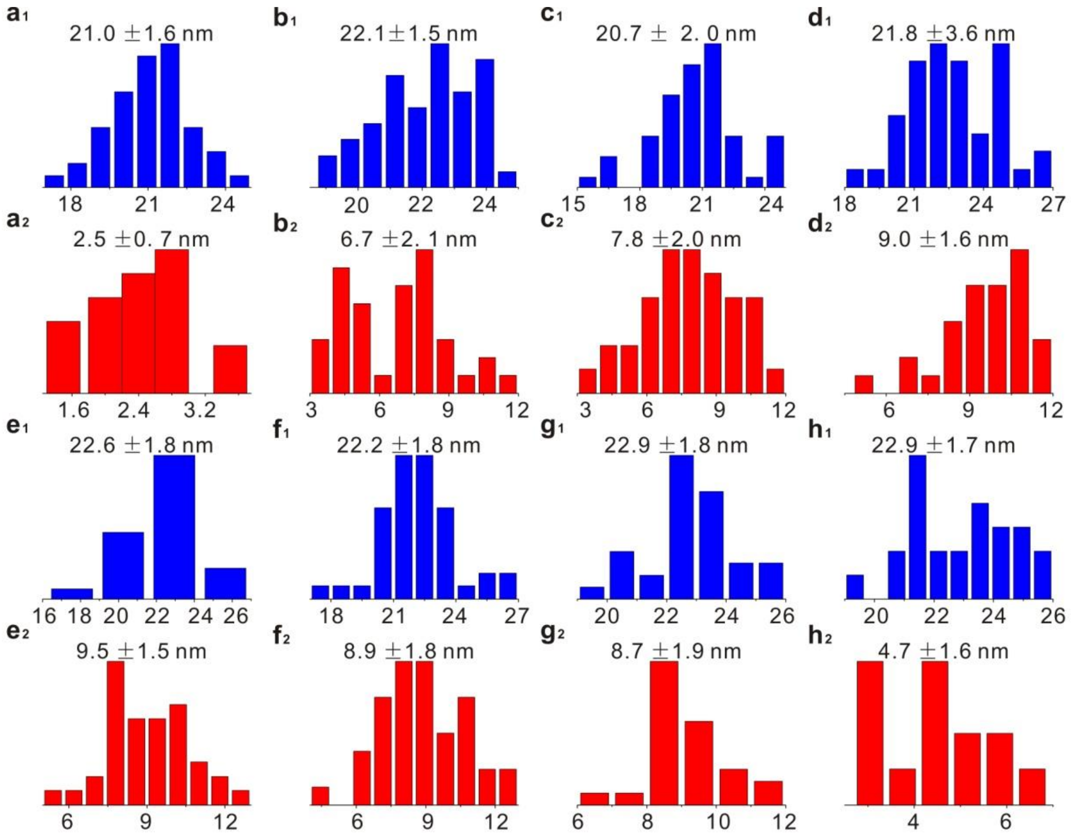

Supplementary Figure 11. Particle size (x_1) and pore size (x_2) distribution of hollow Pd nanoparticles obtained with varied molar ratios of N_2 : O_2: (a) 90 : 10, (b) 85 : 15, (c) 75 : 25, (d) 70 : 30, (e) 60 : 40, (f) 40 : 60, (g) 20 : 80, (h) 0 : 100.

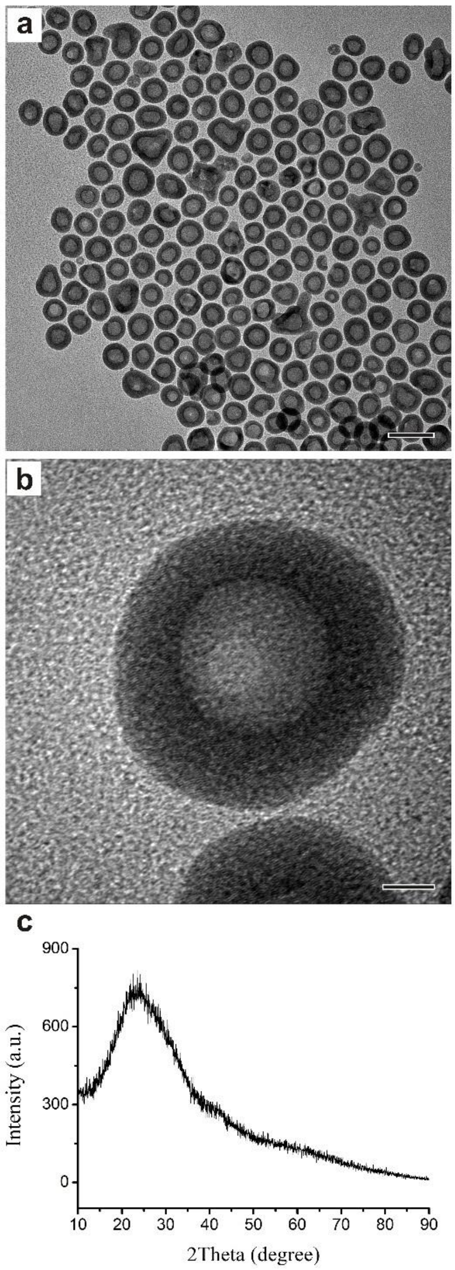

Supplementary Figure 12. Characterization of the amorphous H-Pd-1-P intermediates. (a) TEM image, (b) HRTEM image, and (c) XRD pattern. Scale bar in (a) is 50 nm. Scale bar in (b) is 5 nm.

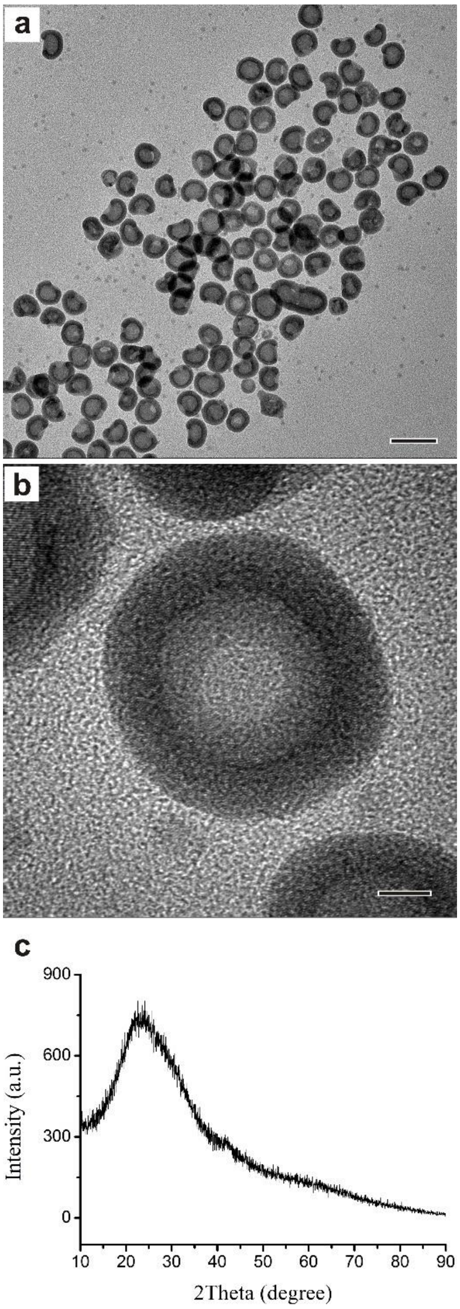

Supplementary Figure 13. Characterizations of the amorphous H-Pd-2-P intermediates. (a) TEM image, (b) HRTEM image, and (c) XRD pattern. Scale bar in (a) is 50 nm. Scale bar in (b) is 5 nm.

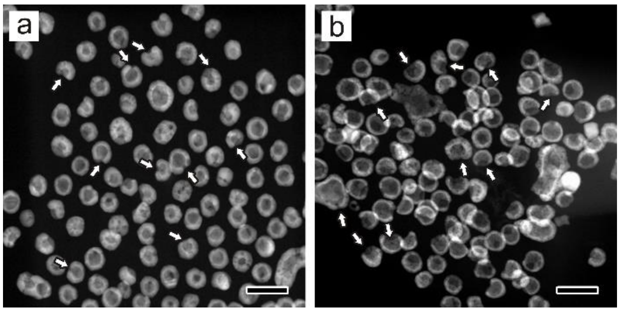

Supplementary Figure 14. HAADF-STEM images of hollow Pd nanoparticles: (a) H-Pd-2, (b) H-Pd-3. Scale bars in (a, b) are 50 nm.

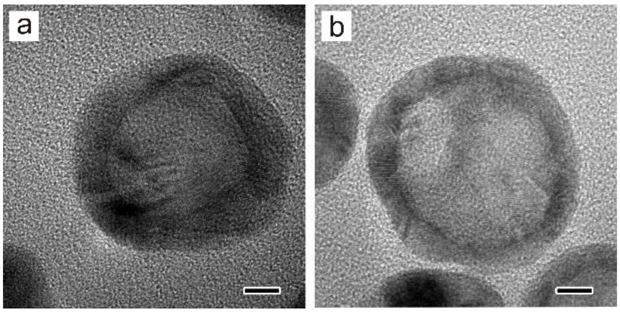

Supplementary Figure 15. HRTEM images of hollow Pd nanoparticles: (a) H-Pd-2, (b) H-Pd-3. Scale bars in (a, b) are 5 nm.

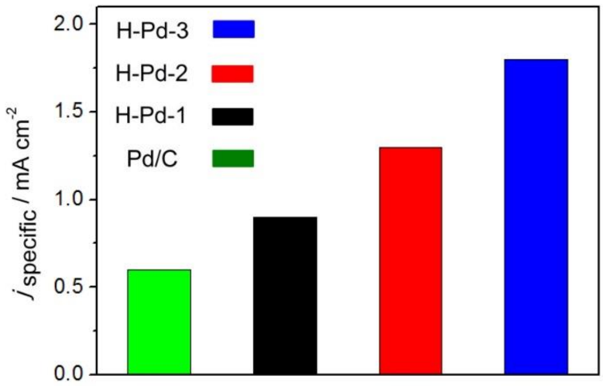

Supplementary Figure 16. Specific activities of the catalysts.

Sample	Pd-P intermediates	Solid Pd nanocrystals	Hollow Pd nanocrystals
Pd : P	1:1.98	8.0:1	8.8:1

Supplementary Table 1. ICP – MS determined content of Pd and P of different samples

Supplementary Methods

Molecular dynamics simulation details

In our simulation, the equation of motion was integrated using the velocity Verlet algorithm with a time step of 0.5 fs, with free boundary conditions applied in all three orthogonal directions. The system was first relaxed in the canonical (NVT) ensemble using the Nose-Hoover thermostat for 5×106 time-steps at 523 K. We then switched to the microcanonical (NVE) ensemble for a further 108 time-steps to perform diffusivity calculations. In order to generate the P concentration gradient as that observed in our experiments, we removed one P atom at the outermost layer per 100 time-steps. The Pd atoms of outermost layer were fixed to maintain the spherical shape. We use the classical embedded-atom-method (EAM),[1-3] in which the total potential energy Etot is written as

$$E_{tot} = \sum_{\mathrm{i}} F_i\left(\rho_{h,i}\right) + \frac{1}{2}\sum_{\mathrm{i,j(\neq i)}} \phi_{ij}\left(r_{ij}\right) \quad (1)$$

$$\rho_{h,i} = \sum_{\mathrm{j(\neq i)}} f_j^{\alpha}\left(r_{ij}\right) \quad (2)$$

where $\rho_{h,i}$ is the electron density around atom i; f_j is the electron density around atom j as a function of the inter-atomic distance r_{ij}, $F_i(\rho_{h,i})$ is the energy to embed atom i into the electron density $\rho_{h,i}$ and $\emptyset ij$ is a two-body central potential between atoms i and j.

$$\phi(r) = A\exp[-\alpha(\frac{r}{r_e} - 1)] - B\exp[-\beta(\frac{r}{r_e} - 1)] \quad (3)$$

where r_e is the equilibrium spacing between the nearest neighbors, A, B, α, and β are parameters. The electron density and embedding energy function can be expressed as

$$\mathrm{f(r)} = \mathrm{f_e}\mathrm{e}^{-\gamma(\frac{\mathrm{r}}{\mathrm{r_e}}-1)} \quad (4)$$

$$\mathrm{F(\rho)} = \mathrm{F_1}\rho^{\mathrm{F_{exp}}} + \mathrm{F_0} \quad (5)$$

The parameter values used in our simulations (listed in Table S2) were chosen by fitting to ab-initio calculated total energy as a function of interatomic spacing. Our ab-initio calculation was implemented in the Vienna Ab initio Simulation Package (VASP).[4,5]

	Pd	P	Pd-P
r_e(A)	2.62	2.65	2.5
f_e(eV)	0.0026	0.0024	-
A	47.4124	2.5215	3.1234
B	46.9145	7.8049	7.8447
α	7.5340	7.3456	8.4948
β	7.3531	2.5971	3.2462
γ	5.51	5.0	-
F_0(eV)	-0.4	-0.4	
F_1(eV)	-55.0	-37.2	
F_{exp}(eV)	0.60	0.60	

Supplementary Table 2. EAM Parameters used in our simulation of PdP_2.

Diffusion coefficient calculations

We obtained the diffusion coefficients *D* at 523 K by analyzing the mean square displacement (MSD) using the least square method based on the Einstein relation. The *MSD* was calculated as

$$MSD \equiv < \Delta \vec{r}(t)^2 > \equiv \frac{1}{N}\sum_{i=1}^{N}(\vec{r_i}(t) - \vec{r_i}(0))^2 \quad (6)$$

MSD is proportional to the observation time t which tends to be infinity. we measure by convention

$$D \equiv \frac{1}{6}\lim_{t\to\infty}\frac{<[r(t_0+t)-r(t_0)]^2>}{t} \quad (7)$$

The angled brackets indicate an ensemble average has been taken. The ensemble average is an average over all atoms. By recoding the atomic positions as a function of time, we calculated the *MSD* (as shown in Supplementary Fig. 8) and then obtained *D*.

Atomistic concentration calculations

The atomistic diffusion process is strongly correlated with the concentration evolution in the Kirkendall effect. Supplementary Fig. 9 shows the atomistic concentration gradient as a function of simulation time. We find that the evolution of the atomistic concentration gradient is consistent to that of the atomistic diffusion coefficient.

$O_2 : N_2$	1 : 9	1.5:8.5	2 : 8	3 : 7	4 : 6	6 : 4	8 : 2	10 : 0
**Pd : P**	7.5 : 1	7.0 : 1	8.8 : 1	8.4 : 1	7.5 : 1	7.0 : 1	7.2 : 1	7.8 : 1

Supplementary Table 3. ICP-MS determined content of Pd and P of hollow Pd nanocrystals obtained with varied concentration of O_2.

Supplementary References

1. Zope, R. R. & Mishin, Y. Interatomic potentials for atomistic simulations of the Ti-Al system. *Phys. Rev. B* **68**, 024102 (2003).
2. Daw, M. S. & Baskes, M. I. Embedded-atom method: Derivation and application to impurities, surfaces, and other defects in metals. *Phys. Rev. B* **29**, 6443 (1984).
3. Zhou, X. W., Johnson, R. A. & Wadley, H. N. G. Misfit-energy-increasing dislocations in vapor-deposited CoFe/NiFe multilayers. *Phys. Rev. B* **69**, 144113 (2004).
4. Blöchl, P. E. Projector augmented-wave method. *Phys. Rev. B* **50**, 17953 (1994).
5. Kresse, G. & Furthmüller, J. Efficient iterative schemes for ab initio total-energy calculations using a plane-wave basis set. *Phys. Rev. B* **54**, 11169 (1996).
